# Imaging Retrospective Study Regarding the Variability of the Osseous Landmarks for IAN Block

**DOI:** 10.3390/jcm14020636

**Published:** 2025-01-19

**Authors:** Andrei Urîtu, Ciprian Roi, Alexandra Roi, Alexandru Cătălin Motofelea, Ioana Badea, Doina Chioran, Mircea Riviș

**Affiliations:** 1 Department of Anesthesiology and Oral Surgery, “Victor Babes” University of Medicine and Pharmacy, Eftimie Murgu Sq. No. 2, 300041 Timisoara, Romania; andrei.uritu@umft.ro; 2 Department of Anesthesiology and Oral Surgery, Multidisciplinary Center for Research, Evaluation, Diagnosis and Therapies in Oral Medicine, “Victor Babes” University of Medicine and Pharmacy, Eftimie Murgu Sq. No. 2, 300041 Timisoara, Romania; rivis.mircea@umft.ro; 3 Department of Oral Pathology, Multidisciplinary Center for Research, Evaluation, Diagnosis and Therapies in Oral Medicine, “Victor Babes” University of Medicine and Pharmacy, Eftimie Murgu Sq. No. 2, 300041 Timisoara, Romania; alexandra.moga@umft.ro; 4 Department of Internal Medicine, Faculty of Medicine, “Victor Babes” University of Medicine and Pharmacy, 300041 Timisoara, Romania; alexandru.motofelea@umft.ro; 5 Maxillofacial Surgeon, Rivis Dental Clinic, 307160 Dumbrăvița, Romania; badeaioanadaniela@yahoo.com; 6 Department of Anesthesiology and Oral Surgery, Research Center in Dental Medicine Using Conventional and Alternative Technologies, “Victor Babes” University of Medicine and Pharmacy, Eftimie Murgu Sq. No. 2, 300041 Timisoara, Romania; chioran.doina@umft.ro

**Keywords:** mandibular foramen, anesthesia landmarks, IAN block

## Abstract

**Background/Objectives**: The aim of this study is to identify the most accurate and consistent landmarks for determining the precise location of the mandibular foramen (MF) and the mandibular ramus, suggesting appropriate adjustments to anesthesia techniques based on these variations in order to improve the success rate of the inferior alveolar nerve (IAN) block. **Methods**: CT scans of the mandibles from 100 patients were analyzed to measure the distance between the MF and various landmarks, including the sigmoid notch, gonion, posterior and anterior margins of the ramus, temporal crest, and the mandibular ramus height from the condyle to the gonion. The width of the mandibular ramus was also assessed, with correlations made to age and gender. **Results**: The MF was found to be closer to the sigmoid notch (mean = 21.2 mm), *p* = 0.393, than to the gonion (mean = 22.6 mm), *p* = 0.801, and closer to the posterior margin of the ramus (mean = 13.1 mm), *p* = 0.753, than to the anterior margin of the ramus. Additionally, the MF was closer to the temporal crest. Age also influenced the position of the MF, with a posterior and superior movement of the foramen, reducing the distance between the MF and the posterior margin of the ramus as well as the MF and the sigmoid notch (*p* < 0.001). **Conclusions**: A precise understanding of the MF’s location will help dentists and oral and maxillofacial surgeons improve the success of the IAN block, avoid injury to the inferior alveola neurovascular bundle, and minimize surgical complications such as paresthesia, permanent anesthesia, and hemorrhage.

## 1. Introduction

The inferior alveolar foramen or the mandibular foramen (MF) is situated in the center of the medial surface of the mandibular ramus. The inferior alveolar nerve, artery, and vein enter the mandibular ramus through the MF following the path of the mandibular canal. The inferior alveolar neurovascular bundle provides vascularization and innervation for the mandibular bone and teeth from the inferior third molar until midline [1,2]. The mandibular canal begins at the MF and descends obliquely forward along the ramus before entering the mandibular body [3]. When a single canal is present, it extends anteriorly through the trabecular bone and reaches the medial incisor’s alveolus. The canal narrows toward the medial side after being broad and close to the mental foramen [4].

The evaluation of the vertical ramus and MF position has crucial importance in almost every branch of dentistry for diagnosing lesions, planning dento-alveolar surgery, orthognathic surgery–vertical ramus osteotomy, or the need to perform the inferior alveolar nerve anesthesia for different medical procedures such as tooth extraction, endodontic treatment, periodontal surgery [5].

The Spix anesthesia technique is the traditional inferior alveolar nerve block (IANB) on the medial part of the mandibular ramus. This is the most popular nerve block method for the anesthesia of the lingual and inferior alveolar nerves at the same time. The success rate of this intraoral anesthesia approach ranges from 71% to 81% [6,7]. The failure in the case of inferior nerve anesthesia might be explained by the inaccurate location due to the variable positions of the osseous landmarks, such as mandible ramus height, width, and MF localization. Another explanation could be that the anatomical features are not precisely located and the needle positioning is incorrect, which can result in the failure of the IANB. The anesthetic needle’s closeness to the MF determines how well this anesthesia works. The inability to observe the location of the mandibular foramen and its alterations is usually the cause of IANB failures [5,8,9]. If the needle is inserted below the MF, there is no installation of anesthesia of IAN. If the needle is inserted too anteriorly, only the buccal nerve is anesthetized. If the MF is located in an anterior position and the needle is inserted too posteriorly, the facial nerve can be anesthetized.

Our study focuses on the variability of the mandibular foramen position in relation to the limits of the mandibular ramus, taking into consideration the horizontal and vertical dimensions and the vertical ramus width and height, which are all measured on the computer tomograph slides. This study aims to provide valuable information that can be useful for performing dental anesthesia in order to raise the success rate for the IANB, guide orthognathic surgeons during the vertical ramus osteotomy, and update the existing medical information for dental students and dental practitioners.

## 2. Materials and Methods

This study’s goal is to determine which landmarks would offer the most accurate and consistent indications for the MF’s and mandible ramus’ precise location and to offer the appropriate anesthesia technique adjustments to account for these variances.

Our study was approved by the Ethics Committee of “Victor Babeș” University of Medicine and Pharmacy Timisoara (No. 18/1 February 2023), and patients agreed and signed an informed consent form that followed the guidelines of the Declaration of Helsinki.

### 2.1. Patient Recruitment

The patients were included considering the following inclusion and exclusion criteria.

The inclusion criteria are as follows:

- An age of 18–93 years;
- Both genders;
- Patients who have performed a computer tomograph CT examination of the skull.

The exclusion criteria are as follows:

- Minor patients;
- Pregnant patients;
- Patients with mandible fractures;
- Patients with mandible reconstruction or titanium osteosynthesis material;
- Patients with tumors that affect the mandible;
- Patients with contraindications for CT;
- Patients with technical or local artifacts that affect the CT images.

All data were anonymous, except for age and gender.

### 2.2. CT Measurements

All the scans were carried out on a Siemens Somatom Definition Edge (Erlangen, Germany) at 120 kV, with a slice thickness of 0.6 mm (mm) and a 0.4 mm slice increment. The 3D reconstruction images were obtained in OpenRad Cloud (Biotronics3D, London, UK).

**Figure 1:**
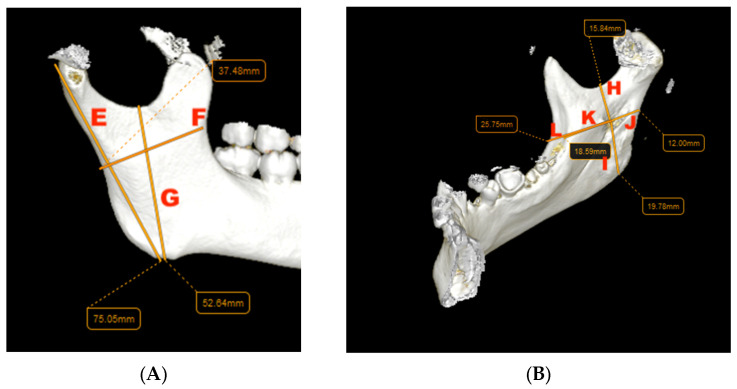
Mandibular measurements on 3D slices: (**A**) external face of the ramus; (**B**) internal face of the ramus.

The linear measurements in millimeters were performed both on 2D slices and 3D reconstruction, as shown in Figure 1, by 2 individual evaluators in order to minimize the bias and errors as follows:

E—mandibular ramus height from condyle to gonion;

F—mandibular ramus width;

G—mandibular ramus height from sigmoid notch to gonion;

H—distance from MF to sigmoid notch;

I—distance from MF to gonion;

J—distance from MF to the posterior border of the ramus;

K—distance from MF to temporal crest;

L—distance from MF to the anterior border of the ramus.

### 2.3. Statistical Analysis

Data were collected using Microsoft Excel and analyzed in RStudio version 4.3.1. The statistical analysis was designed to evaluate the differences in mandibular measurements and their relationship with gender and age based on the collected imaging data. The following approach was applied.

Descriptive statistics, including mean, standard deviation (SD), and range, were calculated for each anatomical measurement (e.g., ramus height, width, and distances from the mandibular foramen to specific landmarks). Age data were summarized separately for males and females, providing the mean, SD, and range for each group.

Prior to inferential analysis, the Shapiro–Wilk test was used to assess the normality of the data for each variable, determining if parametric tests were appropriate. A priori power analysis was conducted to ensure this study was adequately powered for detecting differences in key mandibular measurements. The mandibular foramen–sigmoid notch distance was selected as the primary outcome variable for the power calculation due to its critical relevance in determining the success of inferior alveolar nerve blocks and its statistical evaluation in gender comparisons. For this analysis, the mean difference in the mandibular foramen–sigmoid notch distance between males and females (21.9 mm vs. 20.2 mm) was used, with a pooled standard deviation of 2.85 mm. The calculated effect size (Cohen’s d = 0.596) indicated a medium effect. Assuming an alpha level of 0.05 and a male-to-female ratio of 61:39, a total sample size of 95 participants was required to achieve sufficient power. With 100 participants included in this study, the sample size was deemed adequate for detecting medium-sized effects.

To compare anatomical measurements across gender groups (male vs. female), an Independent Samples *t*-test was performed for normally distributed data. For variables that did not meet the normality assumption, the Mann–Whitney U test was used as a non-parametric alternative.

Patients were stratified into three age groups: 18–30 years, 30–60 years, and >60 years. One-Way Analysis of Variance (ANOVA) was conducted to assess differences in mandibular measurements across these age groups. For variables that did not meet the homogeneity of variance assumption (tested using Levene’s test), Welch’s ANOVA was applied.

When ANOVA indicated significant differences among age groups, Tukey’s Honestly Significant Difference (HSD) test was used for post hoc comparisons to identify specific group differences. To ensure measurement consistency, the Intraclass Correlation Coefficient (ICC) was calculated for the measurements obtained by the two evaluators, with an ICC value >0.75 indicating good reliability.

A significance level of 0.05 was used for all statistical tests. All analyses were performed using R software (Version 4.3.1), with a *p*-value < 0.05 considered statistically significant.

## 3. Results

A total of 39 participants were females (39.0%). The mean age for males was 47.4 years (SD = 17.9), and for females, it was 63.1 years (SD = 21.5), with an overall mean of 53.5 years (SD = 20.7) (*p* < 0.001). Age ranged from 18 to 83 years in males and 22 to 93 years in females.

Table 1 displays the mean and standard deviation of the mandibular foramen position on the internal face of the ramus. As the results show, the mandibular foramen is located, in all of the cases included in this study, closer to the gonion than to the sigmoid notch. Regarding the anterior to posterior position, the mandibular foramen is not located in the center of the ramus but is closer to the posterior margin of the ramus.

Mean distances for the ramus height (condyle to gonion) were 62.2 mm (SD = 5.7) for males and 55.1 mm (SD = 4.6) for females, with an overall mean of 59.4 mm (SD = 6.3) (*p* < 0.001). The range was 50.2–75.0 mm for males and 43.3–64.8 mm for females.

For the ramus width, the mean was 30.4 mm (SD = 5.4) in males and 27.0 mm (SD = 4.1) in females, with an overall mean of 29.0 mm (SD = 5.2) (*p* = 0.001). The ranges were 19.3–48.6 mm for males and 18.0–41.1 mm for females.

The mean distance from the sigmoid notch to the gonion was 48.9 mm (SD = 6.4) in males and 43.1 mm (SD = 4.3) in females, with an overall mean of 46.7 mm (SD = 6.3) (*p* < 0.001). The ranges were 24.9–61.7 mm for males and 26.1–51.5 mm for females.

**Figure 2:**
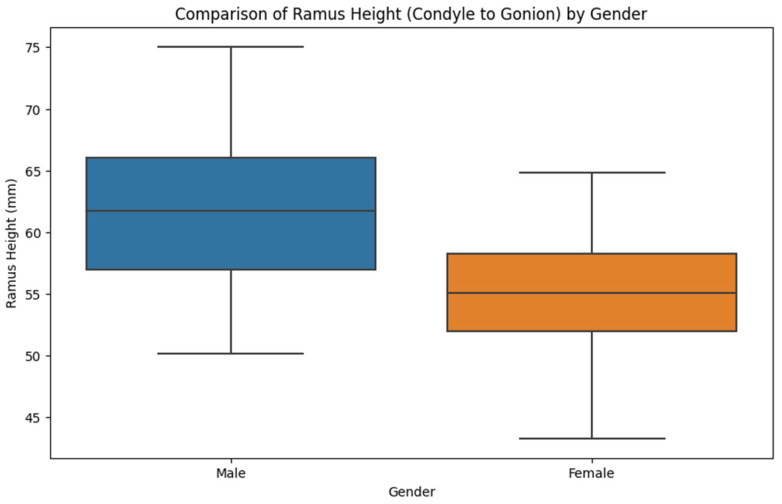
Comparison of ramus height by gender.

Mean distances across gender groups for ramus height (condyle to gonion) were different: males had a mean of 62.2 mm (SD = 5.7) and females 55.1 mm (SD = 4.6), with an overall mean of 59.4 mm (SD = 6.3) (*p* = 0.001). The range was 50.2–75.0 mm for males and 43.3–64.8 mm for females, as shown in Figure 2.

For the age variable, the mean age of the participants was 47.4 years (SD = 17.9) in males and 63.1 years (SD = 21.5) in females, with an overall mean of 53.5 years (SD = 20.7) (*p* < 0.001). Age ranged from 18 to 83 years for males and from 22 to 93 years for females.

As shown in Table 2, male patients compared to female patients have a higher ramus height measured from the condyle to gonion, a higher ramus width, and higher sigmoid notch to gonion dimensions.

**Figure 3:**
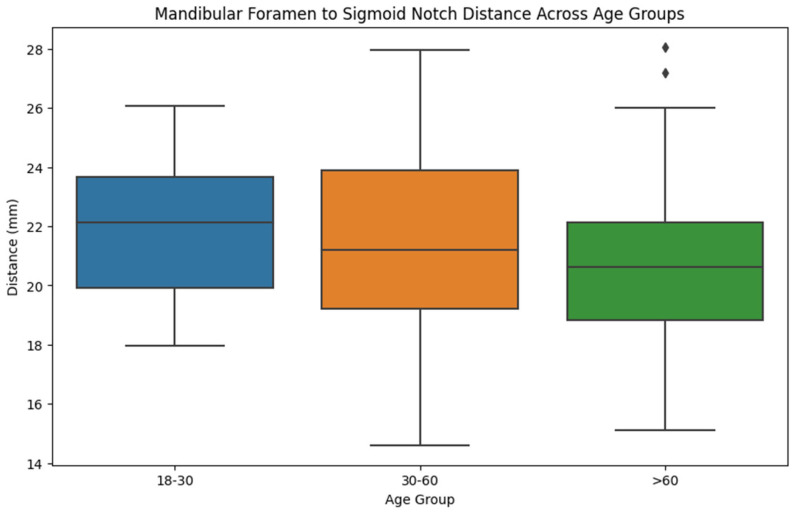
Mandibular foramen to sigmoid notch distance across the age groups.

As shown in Table 3 and Figure 3, the position of the mandibular foramen is influenced by age and is closer to the sigmoid notch and the posterior ramus of the mandible. The relation between the mandibular foramen and gonion, temporal crest, and anterior border of the mandible remains slightly the same when the age increases.

Mean distances across age groups were similar: 21.8 mm (18–30 years), 21.4 mm (30–60 years), and 20.8 mm (>60 years), with an overall mean of 21.2 mm (*p* = 0.393). For the gonion distance, mean values were 22.3 mm, 22.9 mm, and 22.3 mm, with an overall mean of 22.6 mm (*p* = 0.801).

Mean distances to the posterior margin were 13.4 mm, 13.3 mm, and 12.9 mm, with a mean of 13.1 mm (*p* = 0.753). For the temporal crest, the means were 11.9 mm, 13.0 mm, and 12.1 mm, with a total mean of 12.5 mm (*p* = 0.148).

Anterior margin distances were consistent: 18.0 mm, 18.5 mm, and 18.1 mm, with a mean of 18.3 mm (*p* = 0.727). No significant differences were found across age groups (*p* > 0.05), suggesting stable mandibular foramen positioning.

**Figure 4:**
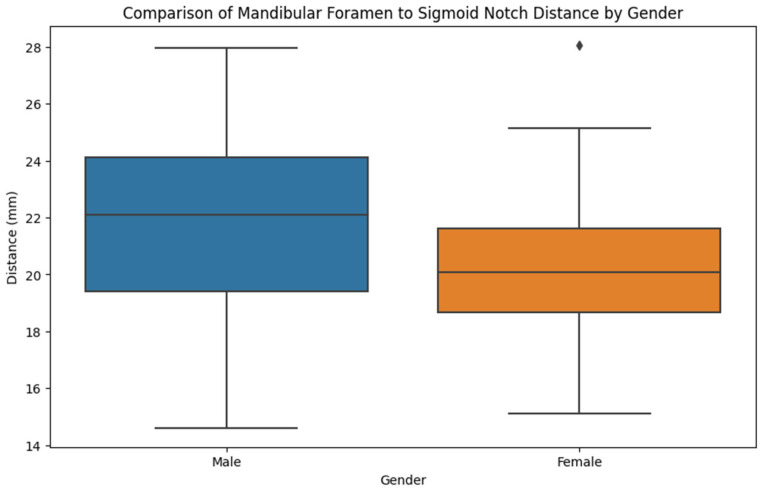
Mandibular foramen to sigmoid notch distance by gender.

Figure 4 presents the correlations between gender and different distances from the mandibular foramen to points located on the internal face of the ramus. In male patients, the distance from the mandibular foramen and sigmoid notch, gonion, posterior margin of the ramus, and temporal crest is higher than in the case of female patients. The distance between the mandibular foramen and the anterior ramus of the mandible is slightly the same in male and female patients.

The mean distance from the mandibular foramen to the sigmoid notch was 21.9 mm (SD = 3.0) in males and 20.2 mm (SD = 2.7) in females, with an overall mean of 21.2 mm (SD = 3.0). Ranges were 14.6–28.0 mm for males and 15.1–28.1 mm for females (*p* = 0.008), showing a significant difference, with males having a longer distance.

For the mandibular foramen to gonion distance, males had a mean of 24.2 mm (SD = 4.1) and females 19.9 mm (SD = 3.2), with an overall mean of 22.6 mm (SD = 4.3). The ranges were 15.2–33.2 mm for males and 14.5–26.2 mm for females (*p* < 0.001), indicating a significantly greater distance in males.

The mean distance to the posterior margin of the ramus was 13.7 mm (SD = 2.3) for males and 12.3 mm (SD = 2.7) for females, with an overall mean of 13.1 mm (SD = 2.5). Ranges were 9.1–19.1 mm for males and 7.7–22.0 mm for females (*p* = 0.009), reflecting a significant difference, with males having a longer distance.

No significant differences were noted for distances to the temporal crest (males: 12.6 mm, females: 12.3 mm, *p* = 0.630) or to the anterior margin of the ramus (males: 18.2 mm, females: 18.3 mm, *p* = 0.826), suggesting similar values between genders.

Overall, significant gender differences were present for most measurements, with males having consistently longer distances, except for the temporal crest and anterior margin, where measurements were similar between genders.

## 4. Discussion

This study demonstrates that the upper position of the MF is closer to the sigmoid notch and the posterior margin of the ramus; our patient measurements show a longer distance from the MF to the anterior border of the ramus. In male patients, the distance from the mandibular foramen and sigmoid notch, gonion, posterior margin of the ramus, and temporal crest was higher than in the case of female patients.

The inferior alveolar nerve block anesthesia is used in every branch of dentistry to achieve a pain-free treatment for the patient. Accurate anesthesia is obtained only when the technique landmarks are accurately determined and the anesthetic substance is deposited as close as possible to the nerve. The mandibular foramen’s exact position needs to be evaluated also prior to orthognathic surgeries, as vertical ramus osteotomy, sagittal split or inverted, and L” osteotomies are important in order to protect the inferior alveolar bundle. The location of the mandibular foramen is crucial for radiotherapists while planning radiation therapy as well as for preventing problems, like bleeding and paresthesia, which are complications of oral surgery procedures.

When a local anesthetic block of the inferior alveolar nerve is necessary, numerical information regarding the mandibular foramen’s location is crucial. The only non-invasive technique currently used for diagnostic and treatment planning of major mandibular surgeries is radiology. Without this paraclinical investigation, an old method was used to assess the position of the mandibular foramen during the IAN block by performing a bi-digital measurement of the thickness of the mandibular ramus. This method is based on the empiric fact that MF would be positioned in the middle of the mandible ramus.

In the present study, our results show that MF is closer to the sigmoid notch mean value of 21.2 mm than the gonion mean value of 22.6 mm. Our results confirm the findings of J. Keros et al.’s study, where the distance between the mandibular notch and mandibular foramen (MF) was 21.3 [10]. Compared to Shalini’s study, where the mean distance from the mandibular notch to the inferior end of the mandibular foramen was 22.5 ± 0.5 mm and the mean distance from the inferior end of the mandibular foramen to the base of the ramus (MF-MB) was 28.44 ± 0.65 mm, our results reinforce the idea that MF is closer to the sigmoid notch [1]. On the other hand, Prajna et al. stated that the MF is located at 22.70 ± 3 mm (right side) and 22.27 ± 2.62 mm (left side) from the mandibular notch, which is slightly higher compared to our study [11].

Regarding the antero-posterior position of the MF, our results demonstrate the fact that MF is closer to the posterior margin of the ramus with a mean value of 13.1 mm than the anterior margin of the mandible ramus (mean value = 18.3 mm). These results compare with the literature findings, where the distance between the anterior ramus ridge and mandibular foramen (MF) was larger in patients with successful anesthesia (19.37), or compared to Mbajiorgu’s study, where the mean distance from the anterior border of the mandibular ramus to the anterior margin of the mandibular foramen was 18.95 ± 0.41 mm [10,12]. Compared to Prajna’s findings, where the average distance of MF from the anterior border of the mandibular ramus was 15.72 ± 2.92 mm (right side), 16.23 ± 2.88 mm (left side), our mean results showed a longer distance from MF to the anterior border of the ramus. Concerning the distance from MF to the posterior border of the ramus, the mean distance was 13.29 ± 1.74 mm (right side) and 12.73 ± 2.04 mm (left side), and the results are comparable to our findings [11]. If we palpate the temporal crest as a landmark for the IAN block, then MF is closer to this anatomic point mean value of 12.5 mm.

In our study, age influences the position of the MF by the posterior and superior movement of the foramen closer to the posterior margin of the ramus and sigmoid notch, decreasing the distance between the MF and posterior margin of the ramus and between the MF and sigmoid notch. These results counter the study conducted by Ashkenazi that demonstrates the anterior movement of MF with aging [13].

As our results demonstrate, male patients compared to female patients have a higher ramus height measured from the condyle to gonion, a higher ramus width, and higher sigmoid notch to gonion dimensions. The gender differences were statistically significant in all of these variables, and all of them were larger in men, which was the same as Pereira’s study [14].

The anesthetic solution must be rapidly distributed from the infratemporal fossa to the mandibular foramen in order to achieve the inferior alveolar nerve block; thus, the needle should be placed next to it to enable the anesthetic to reach the inferior alveolar nerve. To prevent the anesthetic substance from being placed far below or beyond the MF, the needle must be positioned precisely. The parotid gland and the facial nerve may be reached by the anesthetic solution deposited into the posterior area of the mandibular ramus and facial paralysis occurrence [15]. All our measurements can be used for adapting the IAN block technique, starting with the accurate choice of the needle length. The inferior alveolar nerve block by the pterygomandibular technique uses long needles measuring 33 mm and smaller needles measuring 21.5 mm. Using a long needle on a patient with a reduced antero-posterior ramus increases the chance of damaging the facial nerve branches and puncturing the parotid gland capsule. In Peixoto’s study, in more than 95% of the exemplars, the blockage of the alveolar inferior nerve would probably be accomplished with the use of short needles following the pterygomandibular technique [16]. We mention that magnetic resonance microscopy could be more effective for a more precise evaluation of distances from neural tissue [17].

The limitations of this study can be represented by needing to validate these findings through in vivo studies or surgical trials, correlation with soft tissue landmarks for the IAN block, the retrospective design, potential sample selection bias, the generalizability of the findings given the single-center study design, the relatively small number of scans analyzed, and the large interval within the age of the included participants.

## 5. Conclusions

The above findings regarding the IAN block landmarks can help increase the success of inferior alveolar nerve anesthesia. We recommend taking into consideration the upper position of the MF in order to improve the effectiveness of the IANB, which is closer to the sigmoid notch in our study. Additionally, MF is closer to the posterior margin of the ramus; our patient measurements show a longer distance from MF to the anterior border of the ramus.

The precise location of the MF will help oral and maxillofacial surgeons avoid injury of the neurovascular bundle and minimize surgery complications like paresthesia, permanent anesthesia, and hemorrhage.

## Figures and Tables

**Table 1:** Mean and standard deviation of the MF.

Distance, mm	Mean (SD)
Mandibular Foramen–Sigmoid Notch	21.18 (2.94)
Mandibular Foramen–Gonion	22.63 (4.31)
Mandibular Foramen–Posterior Margin of Ramus	13.08 (2.52)
Mandibular Foramen–Temporal Crest	12.47 (2.31)
Mandibular Foramen–Anterior Margin of Ramus	18.30 (2.51)

**Table 2:** The correlation between the mean values of ramus height, width, and sigmoid notch to gonion measures with patients’ gender.

	M (n= 61)	F (n = 39)	Total (n = 100)	*p*-Value
Age				<0.001
Mean (SD)	47.4 (17.9)	63.1 (21.5)	53.5 (20.7)	
Range	18.0–83.0	22.0–93.0	18.0–93.0	
Ramus Height: Condyle to Gonion				<0.001
Mean (SD)	62.2 (5.7)	55.1 (4.6)	59.4 (6.3)	
Range	50.2–75.0	43.3–64.8	43.3–75.0	
Ramus Width				0.001
Mean (SD)	30.4 (5.4)	27.0 (4.1)	29.0 (5.2)	
Range	19.3–48.6	18.0–41.1	18.0–48.6	
Sigmoid Notch to Gonion				<0.001
Mean (SD)	48.9 (6.4)	43.1 (4.3)	46.7 (6.3)	
Range	24.9–61.7	26.1–51.5	24.9–61.7	

**Table 3:** Association between age and mandibular foramen position.

	18–30 (n = 16)	30–60 (n = 43)	>60 (n = 41)	Total (n = 100)	*p*-Value
H: Mandibular Foramen to Sigmoid Notch Distance					0.393
Mean (SD)	21.8 (2.3)	21.4 (3.3)	20.8 (2.9)	21.2 (3.0)	
Range	18.0–26.1	14.6–28.0	15.1–28.1	14.6–28.1	
I: Mandibular Foramen to Gonion Distance					0.801
Mean (SD)	22.3 (4.2)	22.9 (4.2)	22.3 (4.5)	22.6 (4.3)	
Range	15.2–32.0	15.2–33.2	14.5–29.8	14.5–33.2	
J: Mandibular Foramen to Posterior Margin of Ramus Distance					0.753
Mean (SD)	13.4 (2.0)	13.3 (2.2)	12.9 (3.0)	13.1 (2.5)	
Range	9.7–17.3	9.7–19.1	7.7–22.0	7.7–22.0	
K: Mandibular Foramen to Temporal Crest Distance					0.148
Mean (SD)	11.9 (2.1)	13.0 (2.1)	12.1 (2.5)	12.5 (2.3)	
Range	7.8–15.3	8.4–18.6	6.8–18.1	6.8–18.6	
L: Mandibular Foramen to Anterior Margin of Ramus Distance					0.727
Mean (SD)	18.0 (2.9)	18.5 (2.1)	18.1 (2.8)	18.3 (2.5)	
Range	13.7–24.5	13.8–25.8	12.3–25.8	12.3–25.8	

## Data Availability

The data presented in this study are available upon request from the corresponding author.
